# SNPs Occur in Regions with Less Genomic Sequence Conservation

**DOI:** 10.1371/journal.pone.0020660

**Published:** 2011-06-06

**Authors:** John C. Castle

**Affiliations:** Rosetta Inpharmatics LLC, a wholly owned subsidiary of Merck & Co., Inc., Seattle, Washington, United States of America; The University of Chicago, United States of America

## Abstract

Rates of SNPs (single nucleotide polymorphisms) and cross-species genomic sequence conservation reflect intra- and inter-species variation, respectively. Here, I report SNP rates and genomic sequence conservation adjacent to mRNA processing regions and show that, as expected, more SNPs occur in less conserved regions and that functional regions have fewer SNPs. Results are confirmed using both mouse and human data. Regions include protein start codons, 3′ splice sites, 5′ splice sites, protein stop codons, predicted miRNA binding sites, and polyadenylation sites. Throughout, SNP rates are lower and conservation is higher at regulatory sites. Within coding regions, SNP rates are highest and conservation is lowest at codon position three and the fewest SNPs are found at codon position two, reflecting codon degeneracy for amino acid encoding. Exon splice sites show high conservation and very low SNP rates, reflecting both splicing signals and protein coding. Relaxed constraint on the codon third position is dramatically seen when separating exonic SNP rates based on intron phase. At polyadenylation sites, a peak of conservation and low SNP rate occurs from 30 to 17 nt preceding the site. This region is highly enriched for the sequence AAUAAA, reflecting the location of the conserved polyA signal. miRNA 3′ UTR target sites are predicted incorporating interspecies genomic sequence conservation; SNP rates are low in these sites, again showing fewer SNPs in conserved regions. Together, these results confirm that SNPs, reflecting recent genetic variation, occur more frequently in regions with less evolutionarily conservation.

## Introduction

Single nucleotide polymorphisms (SNPs) are intra-species sequence variation. Conversely, cross-species genomic conservation reflects longer term inter-species evolution. SNPs mark genomic locations where intra-species variability is permissible; fewer SNPs should occur in regions encoding sequence-dependent functions. Similarly, insofar that many functional molecular processes are sequence dependent and behave similarly across species, higher genome sequence conservation should occur in functional regions. Therefore, SNP occurrence rates and genomic conservation should be anti-correlated but similarly delineate functional regions. Indeed, there is an emerging field to actively integrate intra- and inter-genomic variation, with applications ranging from disease to phenotype to functional molecular biology to evolution to crop breeding to conservation [1], [2], [3], [4], [5], [6].

Each step in mRNA-processing relies on specific sequences. After transcription, the spliceosome binds RNA to splice exons into a mature transcript; sequence at the transcript is recognized and polyadenylated. The ribosome scans the mature transcript, starting protein translation at a start codon and ending translation at a stop codon. One pathway for mRNA degradation involves miRNA recognition of sequences in the mRNA 3′ untranslated region (3′ UTR).

The human and mouse genomes were assembled in 2001 [7] [8] and 2002 [9], respectively. The University of California Santa Cruz genome databases [10] provide access to many genome-wide resources, including the genomic locations of mouse and human SNPs and a nucleotide-by-nucleotide cross-species conservation score. The databases also lists the locations of predicted miRNA target locations and human transcription factor binding sites and polyadenylation sites, along with genomic alignments of protein-coding RefSeq (NM) transcripts, including start codons, splice sites, and stop codons.

Here, I integrate these genomic coordinates to determine SNP rates and conservation scores surrounding mRNA processing elements. I expected that, for example, SNPs would occur more frequently in codon position three, the degenerate position, and that conservation would be higher at this position. The results indeed confirm expectations and clearly show that SNP rates and conservation are anti-correlated, that both mark functional elements, and demonstrate the single-nucleotide, sequence-level genetic constraints imposed by protein-coding, by splicing, and by transcript processing.

## Results

**Figure 1:**
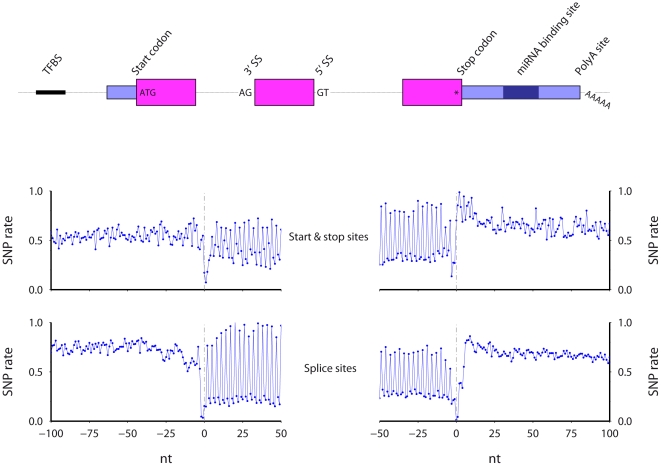
The regions examined, including, protein start sites, 3′ splice sites (3′SS), 5′ splice sites (5′SS), protein stop sites, predicted miRNA binding sites, and polyadenylation sites. Results for mouse start and stop (middle) and splice sites (lower) are shown.

Using mouse and human data available in the UCSC genome databases, I calculate SNP rates and conservation scores at single nucleotide resolution across six mRNA processing regions, for both species (Methods). The SNP rate is the percentage of nucleotides at a given relative position that overlap a SNP. The conservation score at a given relative position is the average inter-species genome conservation score, where the score is taken from the Vertebrate Multiz Alignment & PhastCons Conservation (28 Species) table [11]. Summary diagrams for mouse start, stop, 3′, and 5′ splice sites are shown in Figure 1.

### Start codon (Figure 2)

**Figure 2:**
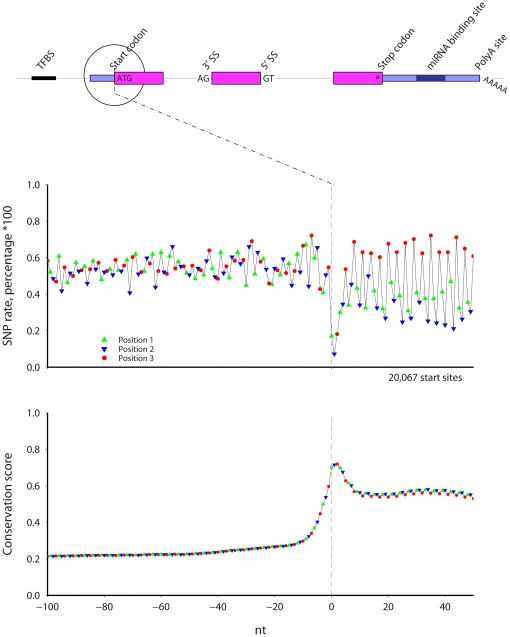
The SNP rate (top) and cross-species conservation (bottom) across mouse protein start sites. Symbol color and shape indicate codon position.

Within the region adjacent mouse protein coding start sites, the SNP rate is lowest and the conservation highest at the start codon. A phase three periodicity in SNP rate and conservation levels occurs after the start codon, with more frequent SNPs and less conservation at codon position three. Within each codon, the majority of positions two, and not positions one, show the lowest SNP rate. After peaking at the start codon to near 0.7, conservation stays high in the coding region at 0.6. The SNP rate is on average lower in positions one and two after the start codon, while the SNP rate in position three is similar before and after the start codon.

### 3′ splice site, phase 0 introns (Figure 3)

**Figure 3:**
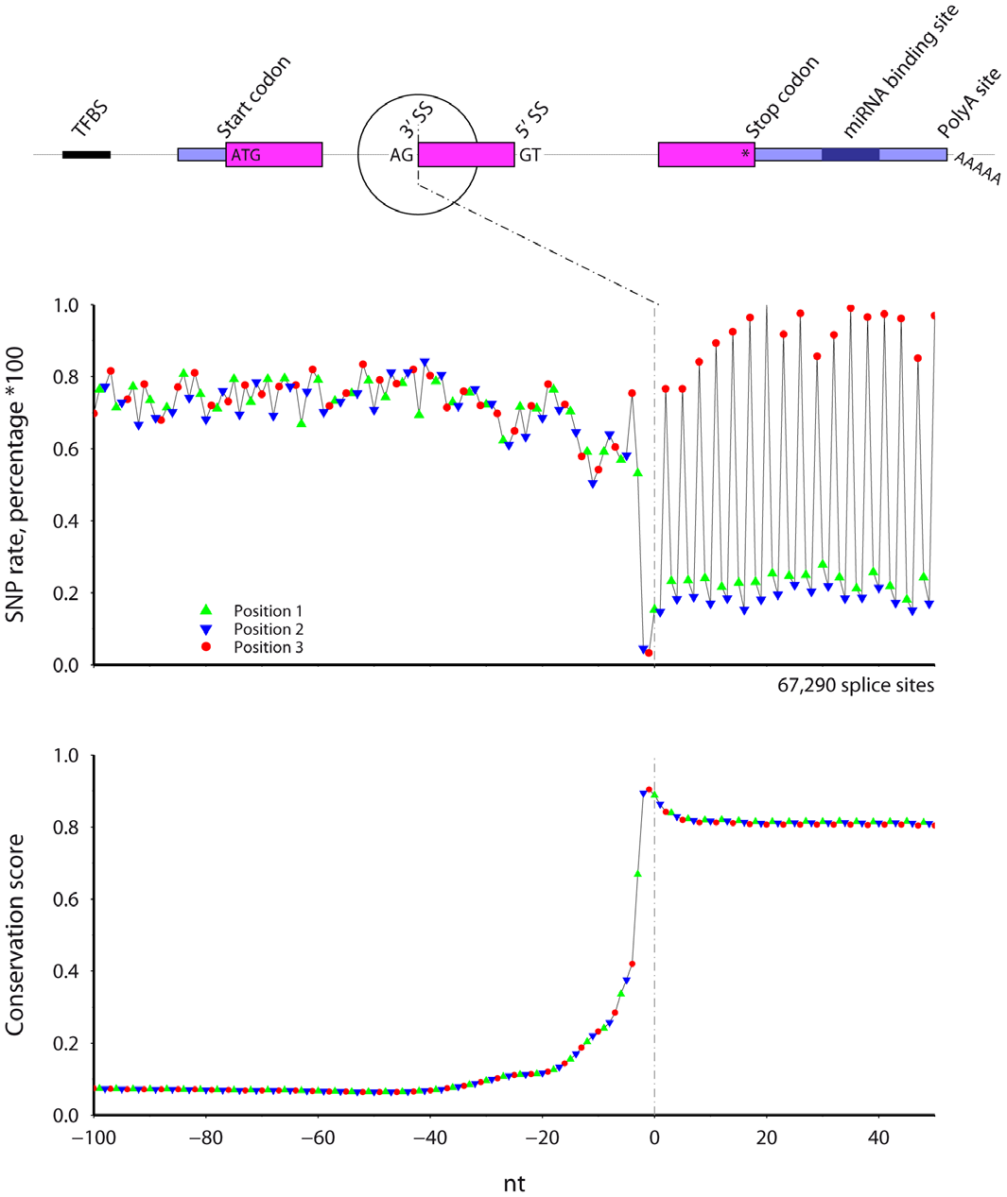
The SNP rate (top) and cross-species conservation (bottom) across mouse 3′ splice sites. Only internal exons longer than 60 nt with phase 0 introns longer than 300 nt were included. Symbol color and shape indicate codon position.

Figure 3 shows the SNP rate and conservation at mouse 3′ splice sites. Introns can be classified according to where they occur relative to the intertwined codons. Introns of phase zero, one, and two represent whether the intron occurs between two codons, after the first nucleotide of a codon, or after the second nucleotide of a codon, respectively. Figure 3 shows results for 3′ splice sites preceded by phase 0 introns. Additionally, for consistency, only exons that are longer than 60 nt and entirely protein coding are included. The SNP rate is lowest and conservation peaks at the splice site AG nucleotides. After the splice site, both the SNP rate and conservation show a clear phase three periodicity. The SNP rate is highest at codon position three. For every codon, the SNP rate is lowest at position 2, lower than the rate for position 1. Conservation stays constant in the exon at over 0.8.

### 5′ splice site, phase 0 introns (Figure 4)

**Figure 4:**
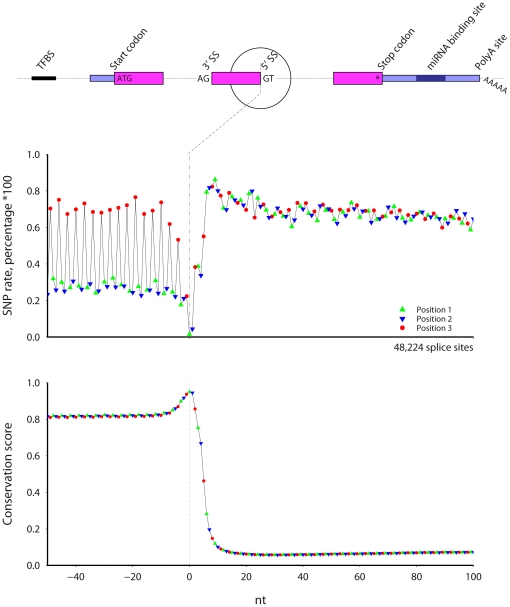
The SNP rate (top) and cross-species conservation (bottom) across mouse 5′ splice sites. Only internal exons longer than 60 nt with phase 0 introns longer than 300 nt were included. Symbol color and shape indicate codon position.

Figure 4 shows results for mouse 5′ splice sites preceding phase 0 introns, using protein coding exons greater than 60 nt long. The SNP rate is lowest and the conservation highest at the splice site GT nucleotides. In the exonic region before the splice site, there is a clear phase three periodicity in both SNP rate and conservation. The SNP rate is highest at codon position three and lowest at position two. The exonic SNP rate at codon positions two and three is low throughout the exon while the position three SNP rate is similar to the intronic rate. Conservation in the exon is high at over 0.8, peaks at the splice site, rapidly decreases, and levels after 15 nt into the intron.

### Splice sites, all intron phases (Figure 5)

**Figure 5:**
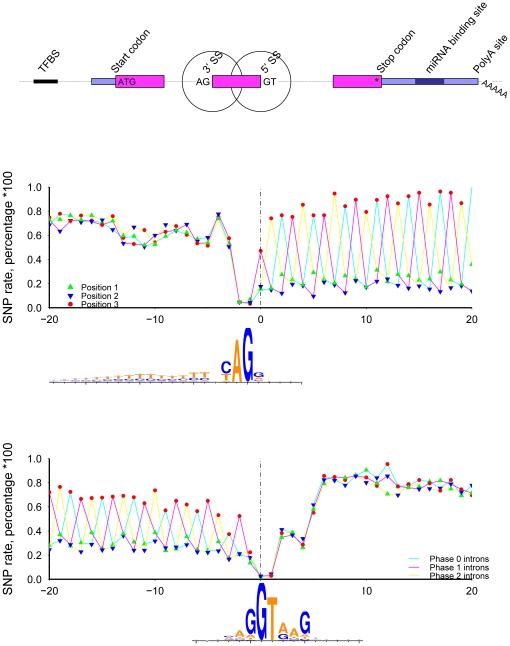
The SNP rate across mouse 3′ (top) and 5′ (bottom) splice sites. Only internal exons longer than 60 nt with introns longer than 300 nt were included. Symbol color and shape indicate codon position. Intron phase is marked by line color. Web-logos were generated using sequences from the phase 0 introns.

The set of mouse RefSeq transcripts contain 50,779, 40,890, and 22,078 phase zero, one, and two introns, respectively, adjacent protein coding exons greater than 60 nt long. Figure 5 shows the SNP rate at 3′ and 5′ splice sites, color coded based on the phase of preceding/following intron. The phase three periodicity in the protein coding region is obvious for all cases, and shifts according to the intron phase. The most SNPs occur at position three and the least at position two. Within intronic regions, all profiles are very similar and no phase three periodicity exists, reflecting the lack of protein coding constraint. Extracting the nucleotide sequences to examine intra-genome variation, I used WebLogo [12] to examine the sequence content and found the expected splice site motifs. Interestingly, at position −4 preceding the 3′ splice site (middle plot), the logo shows no nucleotide preference and, correspondingly, the SNP rate at this specific position is higher than adjacent positions. At position 5 following the 5′ splice site (lower plot), G is the preferred nucleotide; the SNP rate at position 5 is lower than at the neighboring positions.

### Human Splice sites, phase 0 introns (Figure 6)

**Figure 6:**
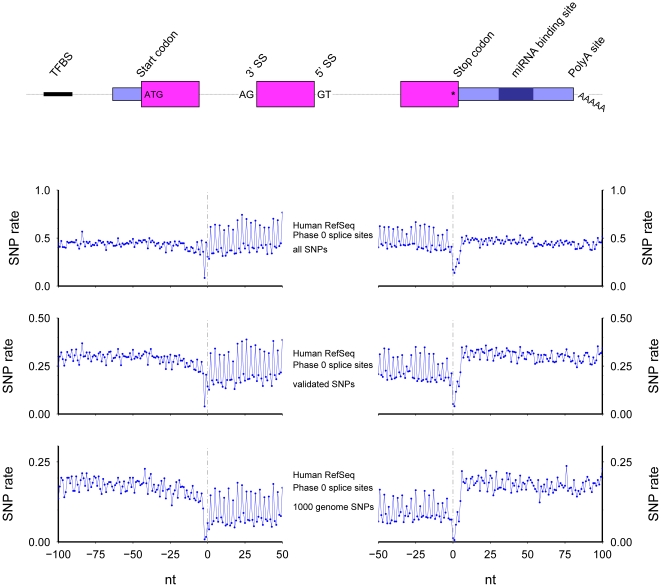
The SNP rate across human 3′ (left) and 5′ (right) splice sites. Only internal exons longer than 60 nt with phase 0 introns longer than 300 nt were included. All SNPs (top), validated SNPs (middle), and 1000 Genomes identified SNPs (bottom) were considered.

The corresponding human genomic regions show results similar to the mouse results (Figure 6). As for mouse, only protein coding exons longer than 60 nt neighboring introns greater than 300 nt long were used. Additionally, three sets of SNPs were examined: all SNPs from dbSNP build 130 [13]; the subset of validated SNPs; and the subset found in the 1000 Genomes Project [14]. The results are similar to the mouse results, including the lowest SNP rates at the splice sites and the period three ringing in protein coding regions. Intriguingly, the main difference between results from the three sets of SNPs is the average difference between exonic and intronic SNP rates, where the SNP rate at exonic codon positions one and two from the validated and 1000 Genomes SNPs is much lower than the intronic rate.

### Stop codon (Figure 7)

**Figure 7:**
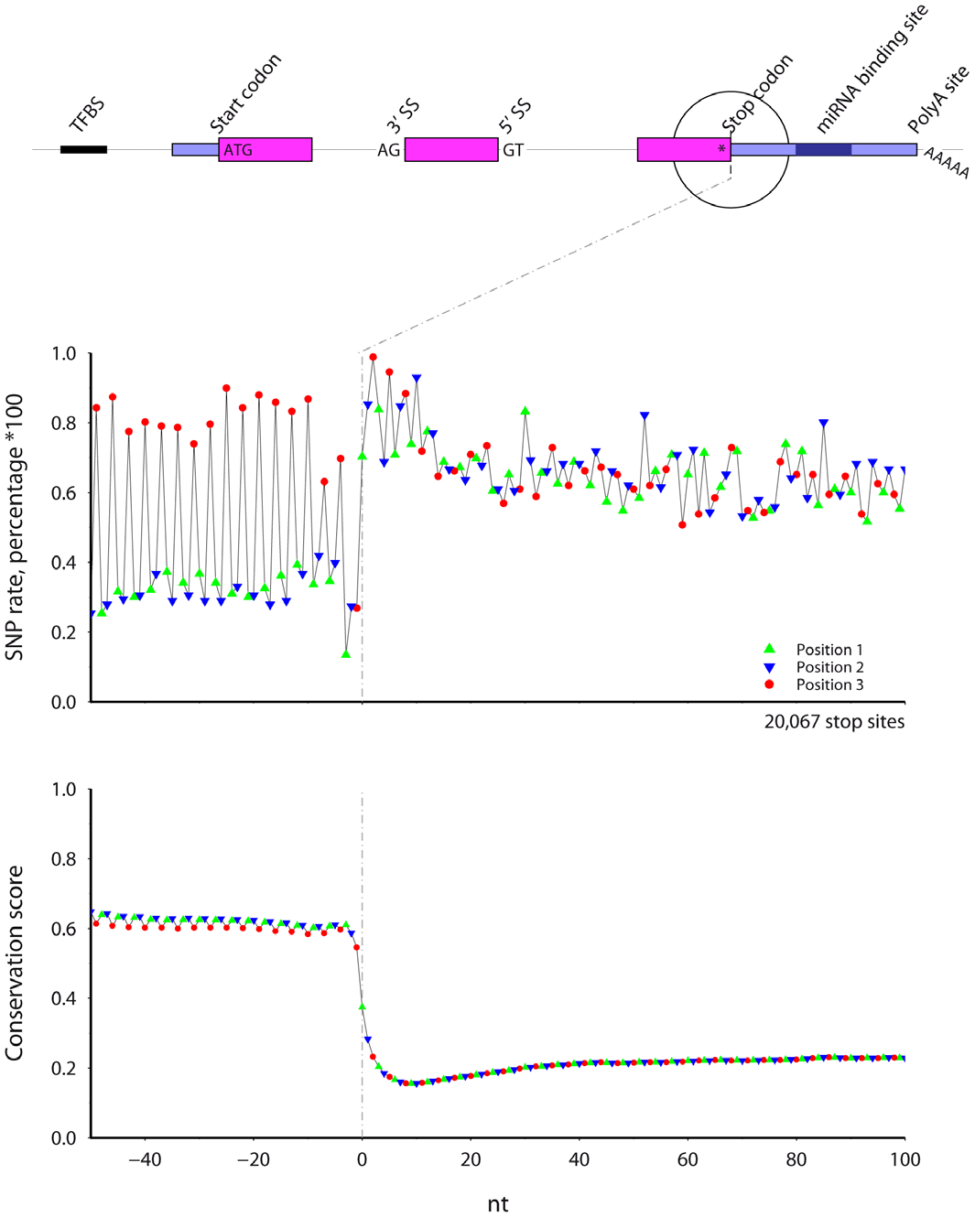
The SNP rate (top) and cross-species conservation (bottom) across mouse protein stop sites. Symbol color and shape indicate codon position.

Near mouse stop codons, the SNP rate is lower and conservation higher before the stop codon. The SNP rate and conservation score display a phase three periodicity before the stop codon. Position three shows a greater SNP frequency and lower conservation; position two shows the lowest SNP rate. The region with both the highest SNP rate and least conservation occurs from 5 to 20 nt after the stop codon, which is found in both human and mouse, suggesting that this region is under less evolutionary control.

### Predicted miRNA binding sites (Figure 8)

**Figure 8:**
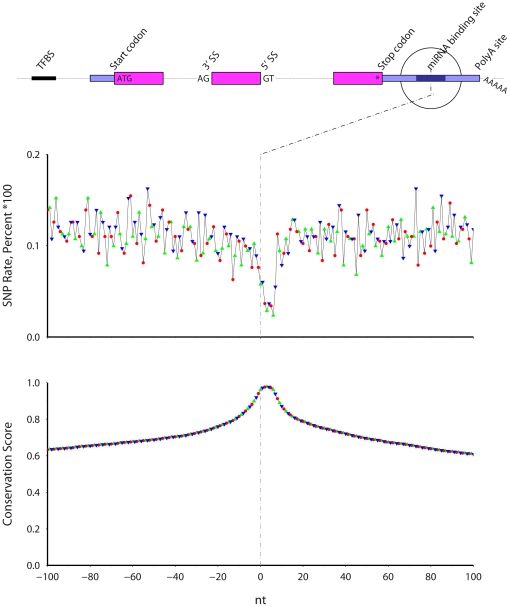
The SNP rate (top) and cross-species conservation (bottom) across predicted mouse miRNA binding sites. Symbol color and shape indicate codon position. The vertical axis extends to 0.2.

TargetScan [15] predicts mouse miRNA 3′ UTR binding sites using genomic conservation and thus genomic conservation should be high at predicted binding locations (Figure 8). I find that the SNP rate is lowest within the predicted miRNA binding locations. The lowest SNP rate occurs across a 5 nt window residing within a larger 8 nt window. The colored symbols in the plot, which marked codon position in previous plots, signify the distance from the 3′ edge, modulus 3, of the miRNA binding site (there are no codons here). As expected, no phase-three periodicity exists in either SNP rate or conservation.

### Transcription termination (Figure 9) and human polyadenylation sites (Figure 10)

**Figure 9:**
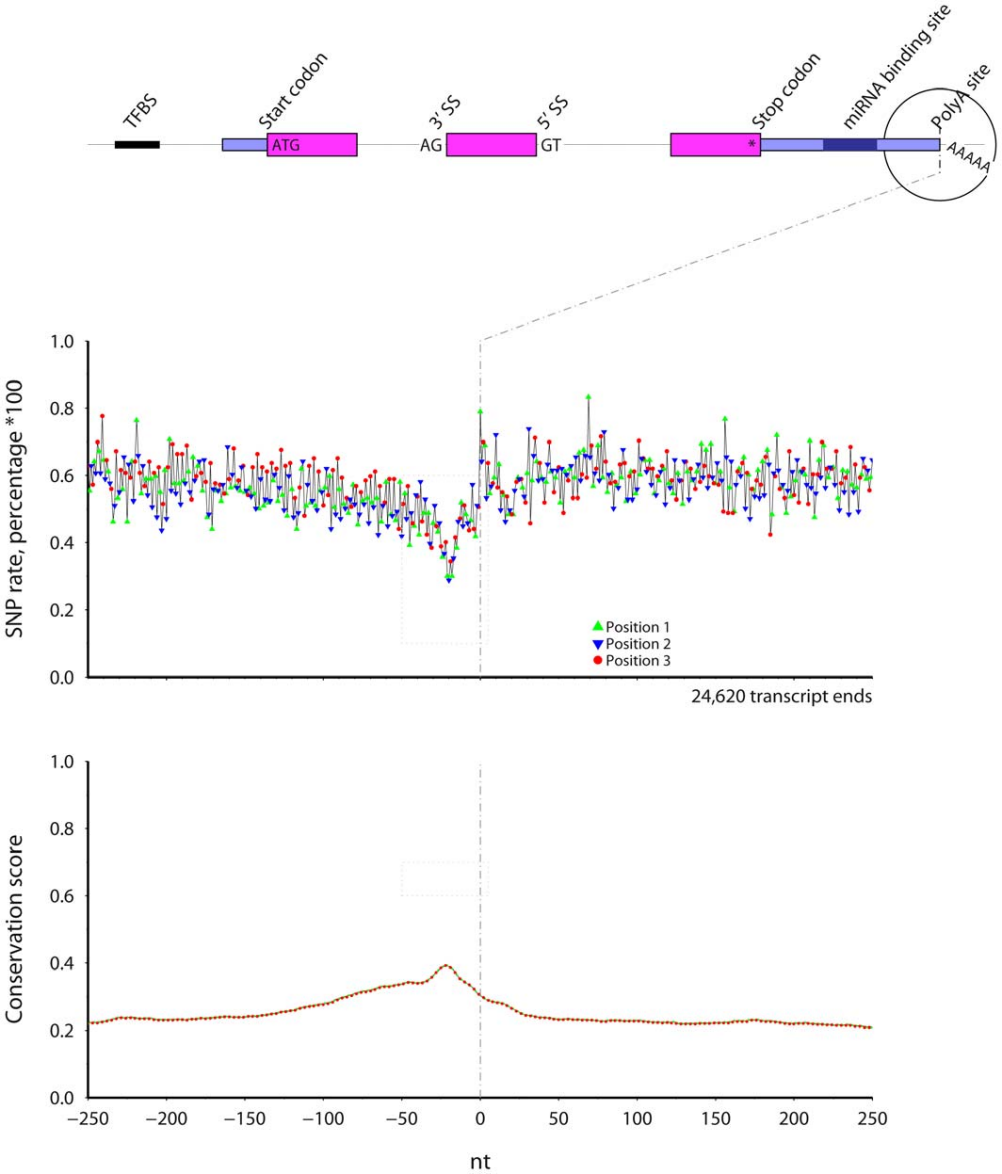
The SNP rate (top) and cross-species conservation (bottom) across mouse transcript 3′ ends. Symbol color and shape indicate codon position.

**Figure 10:**
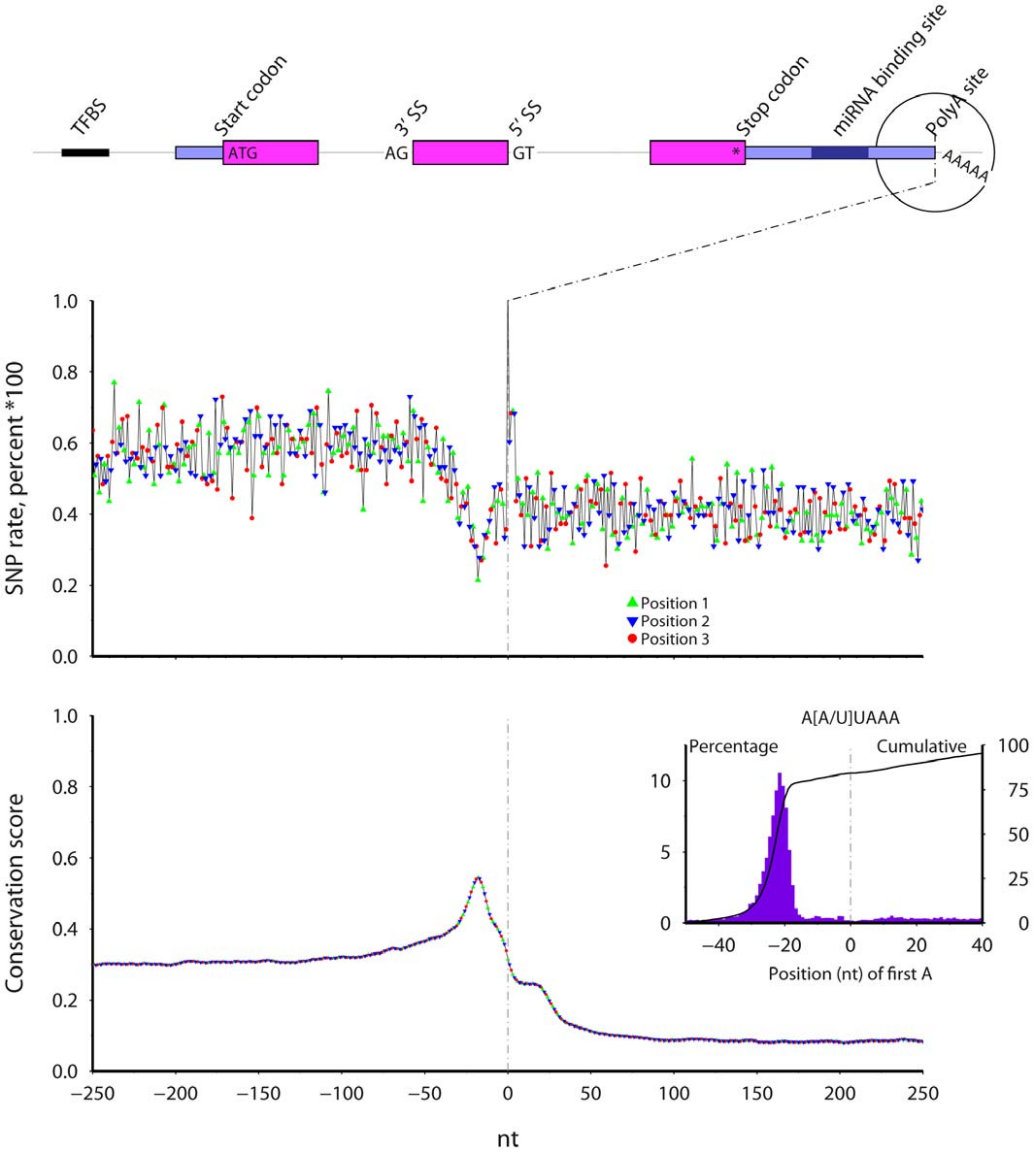
The SNP rate (top) and cross-species conservation (bottom) across human polyadenylation sites. The percentage of sites with the A[A/U]UAAA motif is inset, including the distribution (solid bars) and cumulative level (line).

The UCSC genome databases list transcript 3′ ends for mouse transcripts and predicted polyadenylation sites for human transcripts [16] (Figures 9 and 10). Again, positions 1, 2, and 3 in the plot signify the distance, modulus 3, from the sites (there are no codons here) and, as expected, no phase-three periodicity exists in either SNP rate or conservation. The SNP rate shows a local minimum and the conservation a peak at 20 nt before the polyA site/transcript ends. Before this point, the human 3′ UTR shows higher conservation relative to the region after the polyA site. Beyond the conservation peak, conservation falls but with a 20 nt plateau after the polyA site, followed by a further decrease into the intergenic region (Discussion).

Using the predicted human polyA site coordinates, I extracted the sequence from the UCSC genome databases and searched for the polyA signal motifs AAUAAA and AUUAAA. The motif occurs between 30 and 17 nt before the polyA site (Figure 10 inset) in the majority of the sequences. Thus, the conservation peak at 20 nt before polyA sites likely marks the location of the polyA signal.

## Discussion

Here, I used mouse and human genomic resources to examine SNP rates and interspecies genomic sequence conservation in RNA-relevant regions. The results confirm expectations that the SNP rate and conservation score show anti-correlation: fewer SNPs occur in conserved regions. This is in agreement with our understanding that both processes mark regions under evolutionary conservation: SNPs reflect allowed intra-species genome variation and cross-species conservation demarcates long-term evolutionary stability.

Results from human and mouse datasets are very similar. One difference is that when all human SNPs are considered, the SNP rate in introns is surprisingly lower than in exons (Figure 6, top), However, examination of only high quality SNPs shows SNP rates lower in exons, as expected (Figure 6, middle and bottom). SNPs discovery experiments are often biased based on genomic location: many previous and upcoming studies concentrate on variations (and mutations) in known coding exonic regions [17], further biasing our knowledge of SNPs to those occurring in mRNAs. Thus the appearance of higher SNP rates in exons relative to introns may be a simple sampling bias.

Additional biases may result from the genome of the laboratory mouse genome. Inbred laboratory mouse strains have a genome that is mosaic of two sub-species [18]. The polymorphisms contained in dbSNP are a broad and diverse collection of variations submitted from many contributors. Thus a given SNP may represent either an inter-species variation (e.g., within *Mus musculus domesticus*) or a variation between sub-species (e.g. between *M. m. domesticus* and *M. m. musculus*). The latter represent variation somewhat between a true intra-species polymorphism and cross-species variation.

Furthermore, changes in mutation rate variation could introduce biases in these observations (e.g., [19]). The data could be skewed if, for example, the genome of one species experienced an extreme mutation rate, biasing the genomic conservation; by the erroneous submission to dbSNP of somatic mutations from cancerous cell lines, impacting SNP rates; or by variation in evolution across a genome, such as from variable GC content [20], [21]. The biases likely exist; however, the aggregated results presented here are likely robust to these biases.

Predicted miRNA binding sites [15] incorporate cross-species conservation, along with location relative to transcripts (e.g., in 3′ UTRs) and the underlying nucleic acid sequence, and thus genomic conservation is automatically high. However, as the prediction algorithms do not take SNPs into account, it is satisfying to observe that the SNP rate falls within the predicted mouse binding locations, as has been previously observed for human miRNA binding sites [22], [23]. Indeed, the low SNP rate in these conserved locations further demonstrates that SNPs and interspecies genomic sequence conservation are not independent processes; rather, SNPs occur in less conserved regions. Similarly, SNP rates and conservation around predicted human transcription factor binding sites (TFBSs) shows similar results: SNP rates are low in predicted TFBSs.

The profiles of conservation scores and composite SNP rates delineate established functional elements. Conservation can be used to identify individual elements whereas SNP rates can identify elements when consolidated across the genome. The sharp peak in conservation and the SNP rate trough occurring 20 nt before the polyadenylation site identifies a region highly enriched for the polyA signal AA[U/A]AAA. Motif enrichment, conservation, and the low SNP rate all occur over a narrow range from 30 to 17 nt preceding the polyadenylation site. Variations near 5′ and 3′ splice sites may impact pre-mRNA splicing and can cause disease [24]; correspondingly, high conservation and low SNP rates occur here. A lower SNP rate and higher conservation occurs from +/− 6 nt adjacent the splice sites, similar to previous findings that suggest that this is due to the occurrence of splicing signals [25].

Low genomic variability exists in protein coding regions: SNP rates are lower and conservation higher in all protein coding regions examined. Near the protein start site, conservation is sharply higher precisely at the start site and maintains a high level into the protein coding region. Similar profiles, but in reverse, occur at the protein stop site. However, while the stop codon shows a low SNP rate, conservation at the stop site codon itself is not higher than at previous positions, unlike the spike at the start codon. This may reflect either the stop codon degeneracy or that a mutated stop codon will likely be followed by a second stop codon. Furthermore, compared to the conservation in the internal protein coding exons (e.g., Figure 3), the conservation is lower in the coding regions adjacent the start and the stop codons. Speculatively, this may be due either to a relative decrease in functional importance at the protein N- and C-terminus (unlikely) or simply that our knowledge of the start and stop sites is more uncertain than the coordinates of internal exons, which are clearly defined by bounding AG-GT splice sites.

Additionally, a 15 nt trough in conservation and corresponding high in the SNP rate occur from 5 to 20 nt after the stop site in both mouse and human results. That it occurs in both SNPs and conservation suggests that it is real and, taken at face value, shows that more changes have occurred in the region between the stop codon and 20 nt into the 3′ UTR. Previous work has shown that miRNA binding sites at positioned within the 3′UTR occur at least 15 nt from the stop codon [26], suggesting that the increase in sequence variability in this zone is a result of fewer miRNA binding sites.

Inter-species (conservation), intra-species (SNPs), and intra-genome (webLogos) data all demonstrate the low variation at splice sites. The SNP rate increases almost 3-fold from within the coding exons to the rate at 100-nt into introns. However, the low variability occurs not only in protein-coding regions but also into the adjacent intron, accentuating the need to study variation beyond non-synonymous changes (e.g., [21]). Indeed, the highest conservation and lowest SNP rate occur outside of the protein-coding exons, at the splice sites. This is in line with expectations that while polymorphisms in protein-coding regions could impact amino-acid selection or codon usage, polymorphism and mutations in splicing motifs could deregulate or cause the skipping of entire exons.

Finally, all protein coding regions display a phase three periodicity with increased variability at position three. The periodicity is not observed outside of the protein coding regions, such as adjacent polyadenylation sites. That the SNP rates are highest at codon position three is undoubtedly a result of the degeneracy for amino acid coding at the third position (19 of 22 amino acids are degenerate at codon position 3). Moreover, codon degeneracy is slightly higher at codon position one relative to position two: I correspondingly find a lower SNP rate at the second position. Thus, more SNPs occur in degenerate positions for amino acid encoding, and are thus less likely to have a functional impact [6].

Together, these findings reinforce our understanding of evolution and functional elements in the genome. These include that SNPs occur more frequently in less conserved regions; that both genomic conservation and aggregated SNPs rates can be used to identify functional elements; and that codon position three in protein coding regions is more degenerate.

## Methods

Genomic coordinates were downloaded from UCSC databases [10], including human dbSNP build 130 and mouse dbSNP build 128 [13], human predicted transcription factor binding sites (TFBS) from the HMR Conserved Transcription Factor Binding Sites table [27], mouse and human predicted miRNA target sites from the TargetScan table [15], reported human polyadenylation sites [16], alternative transcriptional element alignments (derived in the UCSC databases from the unpublished txgAnalyse and txGraph programs written by Jim Kent at UCSC), and RefSeq NM transcript and EST sequence alignments. I accessed the nucleotide-specific cross-species conservation scores from the Vertebrate Multiz Alignment & PhastCons Conservation (28 Species) table [11]. As mentioned, the predictions of both TFBS and miRNA binding sites explicitly use cross-species genome conservation as criteria. Polyadenylation site predictions incorporate the coordinates of 3′ ESTs, including the presence of polyA tails. Validated human SNPs are those containing the word “by” in the description (e.g., “by-hapmap”) and “1000 Genome” SNPs are those listing “by-1000genomes”. The SNPs identified in the 1000 Genomes Project are not error-free; however, I expect no systematic error that would impact the presented aggregated SNP rates. Transcript alignments were performed by UCSC using BLAT.

For the human predicted polyadenylation sites, I selected only those polyadenylation sites falling within 100 nucleotides of the 3′ terminus of a RefSeq transcript. This excluded alternative polyadenylation sites occurring in the interior of 3′ UTRs and also allowed a strand assignment to each polyadenylation site, based on the RefSeq transcript orientation.

I used RefSeq “NM” transcript alignments which include exon boundaries, transcription start and stop coordinates, coding start and stop coordinates, and intron phase. I required at least 60 nt of coding sequence after and before start and stop codons, respectively. For the 5′ and 3′ splice sites, I included only internal exons longer than 60 nt surrounded by introns greater than 300 nt long.

I examined several mRNA processing regions (Figure 1): protein start codons, internal 3′ splice sites, internal 5′ splice sites, protein stop codons, predicted miRNA binding sites, predicted polyadenylation sites (human), transcript 3′ termini (mouse), and predicted transcription factor binding sites (human), For each region, the algorithm looped over each individual site, counting SNPs and the level of conservation at each position between −500 and +500 nt around the site. Reported values reflect the percentage (SNP rate) and average (genomic conservation) at each position relative to the specific site location. The UCSC genome database also includes the underlying nucleotide sequence, which was used for the WebLogos.
